# Revising the Parallel-Pathways Hypothesis with Time

**DOI:** 10.3389/fnsys.2017.00059

**Published:** 2017-08-17

**Authors:** Albert Tsao

**Affiliations:** Department of Biology, Stanford University Stanford, CA, United States

**Keywords:** LEC, neural population dynamics, episodic memory, mixed selectivity, temporal context

One of the major theories guiding our journey to understanding entorhinal function has been the parallel-pathways hypothesis. Based on the anatomical segregation of entorhinal inputs to the hippocampus between those arising from lateral and those arising from medial entorhinal cortex (LEC/MEC), along with the divergent set of inputs each entorhinal subdivision receives, it was proposed that LEC and MEC fulfilled different functional roles (Burwell, 2000; Witter et al., 2000). Targeted recordings in each subdivision of entorhinal cortex supported this idea of split functions, as LEC cells exhibit poor spatial selectivity, whereas MEC cells exhibit very strong spatial selectivity (Hargreaves et al., 2005). Results from the hippocampus indicating that place cells also utilize separate coding mechanisms for spatial and non-spatial information (Leutgeb et al., 2005) gave rise to a more specific form of the parallel-pathways hypothesis, in which LEC processed the non-spatial “what” component of episodic memories being formed in the hippocampus, while MEC processed the spatial “where” component (Knierim et al., 2006; Eichenbaum et al., 2007). This form of the parallel-pathways hypothesis has been highly informative for guiding experiments over the past decade, but it is not the only lens through which we can ask questions about LEC function. Below I will begin from a different starting point by looking at what current results tell us explicitly about LEC function in order to establish that a critical function of LEC is the formation of higher-order associations, and work my way back to an updated version of the parallel-pathways hypothesis which emphasizes a new functional role for LEC: representing time.

Behavioral studies using various association-based tasks have offered the clearest view of LEC function. In one set of studies, object recognition tasks are used to probe the role of LEC in forming associations involving objects (Wilson et al., 2013a,b). Immediate-early gene (IEG) staining shows that strong LEC activity is driven by engagement in an object-context recognition task that requires the formation of two object-context associations. By comparison, IEG expression when animals are simply exposed to objects is far weaker, suggesting that LEC itself is more involved in forming associations with objects rather than representing the objects themselves. Consistent with this, animals with lesioned LEC have no difficulty performing basic object recognition tasks, but are significantly impaired in forming object-place, object-context, place-context, or object-place-context associations. Along these lines, a related set of LEC lesion studies has shown that LEC is particularly critical for encoding representations of complex sets of stimuli, which may be interpreted as higher-order associations (Van Cauter et al., 2013; Rodo et al., 2017). A different set of studies have demonstrated the necessity of LEC for learning various associations under a trace-conditioning paradigm (Esclassan et al., 2009; Morrissey et al., 2012), while two additional studies have found that LEC is necessary for forming various cross-modal sensory associations (Chen et al., 2013; Boisselier et al., 2014). Thus, across a wide range of experiments, the necessity of LEC for forming high-level associations has been demonstrated.

These behavioral results are consistent with the longstanding view of entorhinal cortex as being a higher-order association area based on its anatomical connectivity (Burwell and Amaral, 1998; Burwell, 2000). However, while many other areas such as parietal and prefrontal cortex also show connectivity indicative of associative function, the formation of associations may be a particularly significant function of LEC. Two recent large-scale anatomical studies have identified LEC as one of the largest connectivity hubs across the entire rodent brain (Zingg et al., 2014; Bota et al., 2015). Moreover, within the areas LEC is anatomically connected to, two of the most significant inputs arise from perirhinal (PER) and ectorhinal cortex, which are each themselves significant hubs of connectivity. Thus, the wide diversity of inputs to LEC suggest that it is uniquely positioned to form higher-order associations. In particular, given that it sits at the top of the associational hierarchy, LEC should bind together the already-high order representations that it receives from perirhinal and other structures into a representation of what is being experienced. This binding can come in the form of associating an object with the place it is found in, or two different sensory stimuli happening across time, and importantly, suggests that the nature of associations formed by LEC may differ from those formed by earlier structures. Whereas PER may be binding together the features of an object into a single representation, LEC is binding these already-compressed representations—the associations being formed are not just cross-modal, but cross categories of representation, e.g., contexts and events happening across time. In total, our current understanding of LEC's anatomical connectivity is consistent with the clearest result from behavioral experiments that a major function of LEC is to generate associations.

To what degree do direct recordings reflect associative function, or provide understanding of how it is carried out? The clearest results have come from experiments in which animals are engaged in behavioral tasks, with LEC neurons developing responses to task cues (Igarashi et al., 2014; Keene et al., 2016). Notably, both experiments use associational tasks containing a spatial component, indicating that LEC is not strictly limited to representing non-spatial information. However, while LEC responses to cues during task performance are consistent with its hypothesized associative function, given that similar associations have been observed to form upstream of LEC (Fiser et al., 2016), the degree to which these results inform us about what is unique to LEC function is limited. The more common approach to studying LEC function through direct recordings has been to probe its responses to various sensory stimuli, such as objects or contexts. The most informative of these experiments have used physical objects placed around the recording environment (Deshmukh and Knierim, 2011; Tsao et al., 2013). During a single recording trial, a sizeable proportion of LEC cells fire around or develop spatial firing fields in the vicinity of these objects. However, across multiple trials, these responses tend to be unstable, and overall, LEC does not appear to generate a stable representation of objects alone. Additionally, manipulations of the objects generally fail to reveal significant insights into LEC function. A very small proportion of LEC cells do develop spatially stable firing fields at locations in which objects have been, and for extremely familiar object-context associations in which the same object was placed in the same location in the same environment across many days of experiments, this activity is context-specific, in agreement with behavioral experiments showing that LEC is critical for object-context associations. However, given how rare these cells are, they likely do not make up the primary mechanism by which associative coding is accomplished in LEC. Overall up to now, experiments using object stimuli have not yielded a clear code for object representation in LEC. Two other studies have examined LEC activity in response to varying contexts. In one experiment using a paradigm which elicits rate-remapping in hippocampus, even though LEC lesions are shown to have an effect on hippocampal rate-remapping, LEC activity itself does not show any clear code for context at the single-cell level (Lu et al., 2013). In another set of experiments using a circular track with highly salient local cues along the track, single LEC cells do not show differential activity across the different parts of the track (Yoganarasimha et al., 2011), even though during experiments rotating the maze relative to constant distal cues, LEC apparently encodes these local cues at the population level (Neunuebel et al., 2013). From these two experiments, it is clear that LEC as a population must be encoding context, but the manner in which this is done is unclear at the single-cell level. Overall, existing LEC recordings have struggled to reveal any clear insights into the computations it carries out, particularly in regard to its associative function.

While behavioral studies of LEC have largely focused on object-related associations, the associations necessary for episodic memory go beyond this single category. In particular, significant questions still remain regarding the mechanisms underlying temporal associations-the integration of “when” with “what”—and this particular form of association may offer a way forward for understanding LEC function. Representation of time within the hippocampal-entorhinal circuit has been observed at two temporal scales: across hours, place cell activity in CA2, and to a lesser extent CA1, becomes significantly decorrelated (Mankin et al., 2012, 2015) while on the order of seconds, cells in CA1, CA3, and MEC encode time by firing at specific points in time (MacDonald et al., 2011; Kraus et al., 2015; Salz et al., 2016). However, these two representations of time do not make up a sufficient system for temporal representations. Time cells have only been observed following learning of a behavioral task, during delay periods of the task, so it is unclear whether they possess the flexibility that a temporal association mechanism would require, given that episodic memories can be formed in one shot. Decorrelated activity in CA2, on the other hand, appears to have the necessary flexibility, as it happens spontaneously. However, it is unclear whether this mechanism covers the entire timescale necessary for episodic memory, as CA2 activity across minutes is still highly correlated. Ideally, time would be represented in a form which was a mixture of these two observations—decorrelated activity on the scale of seconds. Given that the other hippocampal fields and MEC do not show this sort of activity, we shall evaluate whether there is reason to think that LEC takes on this function.

To begin, the integration of time into associative representations would provide a potential explanation for why direct recordings of LEC activity have struggled to provide clear insights into LEC function. The addition of a temporal component to representations of an object-context association, for example, would necessarily create a difference in LEC activity patterns across repeated exposures to the same object-context pairing, which could be interpreted as instability in the representation of the association if one were not expecting time to also be part of the representation. Is there any additional reason to suspect that LEC plays a significant role in associating time with experience? It has been proposed that time can be represented in state-dependent networks through a continuously-changing population state (Howard and Kahana, 2002; Karmarkar and Buonomano, 2007; Buonomano and Maass, 2009). LEC is a prime candidate for implementing this type of de novo population-level encoding of time: the hub-like nature of LEC means that in addition to receiving inputs from various sensory regions, LEC also receives inputs from regions which determine various internal states (Burwell, 2000; Kerr et al., 2007). Input from the amygdala and other structures involved in determining emotional state, for example, implies that for any given point in time, emotional state impacts the overall activity in LEC. Similarly, input from insular cortex and other structures involved in determining hunger state implies that for any given point in time, hunger state impacts the overall activity in LEC. The integration of these states and others, such as motivation, along with a representation of the external world, which itself may be changing across time and is also influenced by attention, could then produce an overall population state which is different for each point in time. The fact that LEC activity is apparently more stable when animals are engaged in a behavioral task is consistent with the idea that internal states impact LEC activity to produce a highly-varying population state, since these internal states would be far more uniform when animals are engaged with familiar tasks. Moreover, the increased consistency in activity appears with learned behavior, when animals are more likely to be engaged in memory recall, as opposed to encoding new memories. Importantly, this mechanism for generating a representation of time would inherently associate the contents of experience with time—the representation of an experience itself is embedded in the population state which is inherently encoding time. This continuously-varying population state could then be used as a representation of time by the hippocampus to generate a more specific representation of time which is used for episodic memory. Episodic memories contain multiple levels of temporal detail (e.g. we can recall that an experience happened many years ago, or we can recall the specific order that events happened during that experience), suggesting that multiple forms of temporal coding may be used. Population-level drift from LEC could be scaled by different degrees in CA2 and CA1 to generate two different scales of time on the order of hours to days. Representation of the fine-scale temporal order of events, on the other hand, may be influenced by the seconds-level variability of LEC activity, which would drive a unique sequence of hippocampal cells to fire for a given experience. This unique sequence could then be consolidated through hippocampal replay, resulting in time cells that would exist within a broader temporal context set by LEC, CA2, and CA1.

To return to the parallel-pathways hypothesis, we might re-form it in terms of associative function: whereas a large body of evidence exists demonstrating that MEC generates a metric representation of space (Moser et al., 2008; Rowland et al., 2016), which may be interpreted as the association of its various inputs into an allocentric representation, LEC forms a different type of association, binding together all the components of experience, including time. The hippocampus can then efficiently integrate these two signals to form episodic memories. Importantly, the representation of experience that LEC forms is not equivalent to episodic memory, as episodic memory exists in the recall of an experience, rather than the actual in-the-moment experience itself. The computations necessary to allow for this recall would be carried out by hippocampus, through its ability to pattern separate/complete and replay experiences, consolidating them into memories, while the representation of the in-the-moment experience, including its temporal context, would be generated by LEC. This revised form of the hypothesis would relax the division of spatial and non-spatial information, as MEC would be predicted to encode non-spatial features such as objects, which may inform its path-integration process (Hardcastle et al., 2015; Keene et al., 2016), while LEC would be predicted to encode spatial information in instances where it relates to experience, which current results from LEC indicate (Deshmukh and Knierim, 2011; Knierim et al., 2013). Most relevant to our understanding of LEC function, this version of the parallel-pathways hypothesis predicts that what appears as unstable activity in single LEC cells is actually a significant feature of the structure.

## Author contributions

The author confirms being the sole contributor of this work and approved it for publication.

### Conflict of interest statement

The author declares that the research was conducted in the absence of any commercial or financial relationships that could be construed as a potential conflict of interest.
